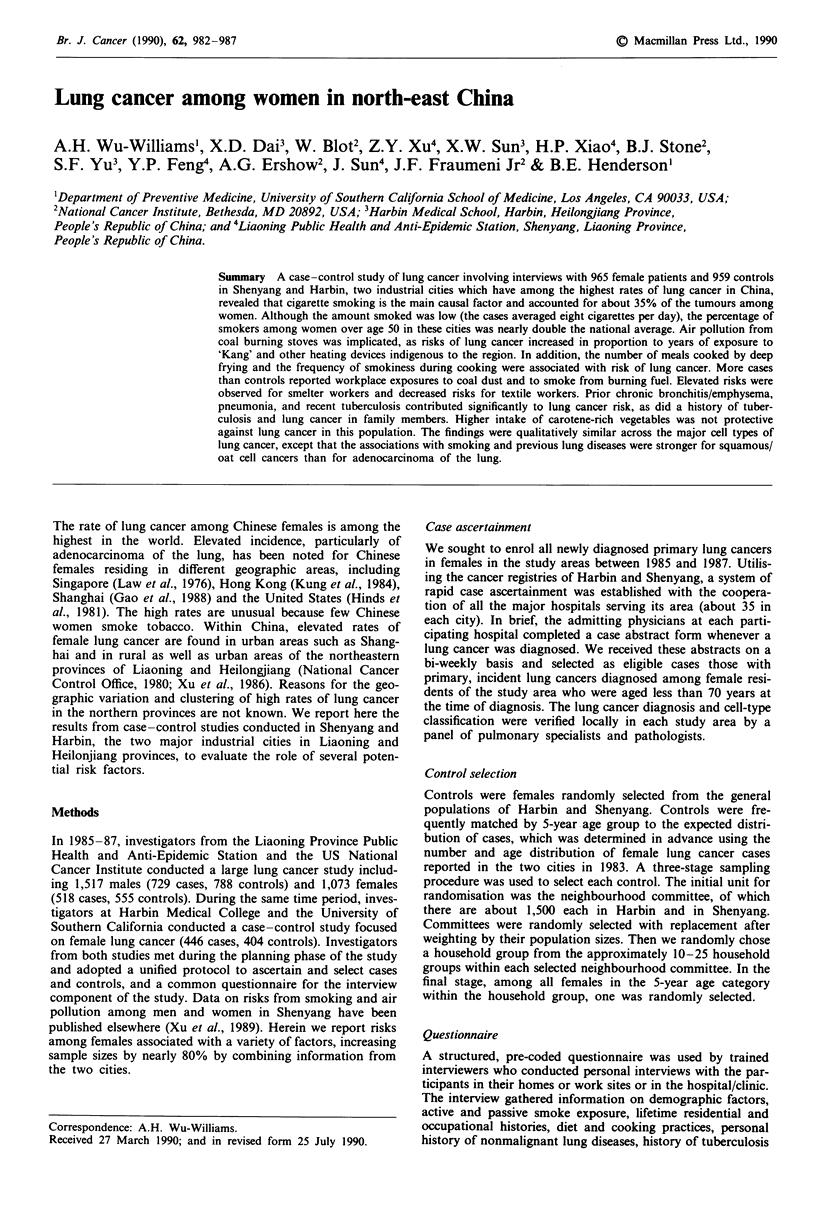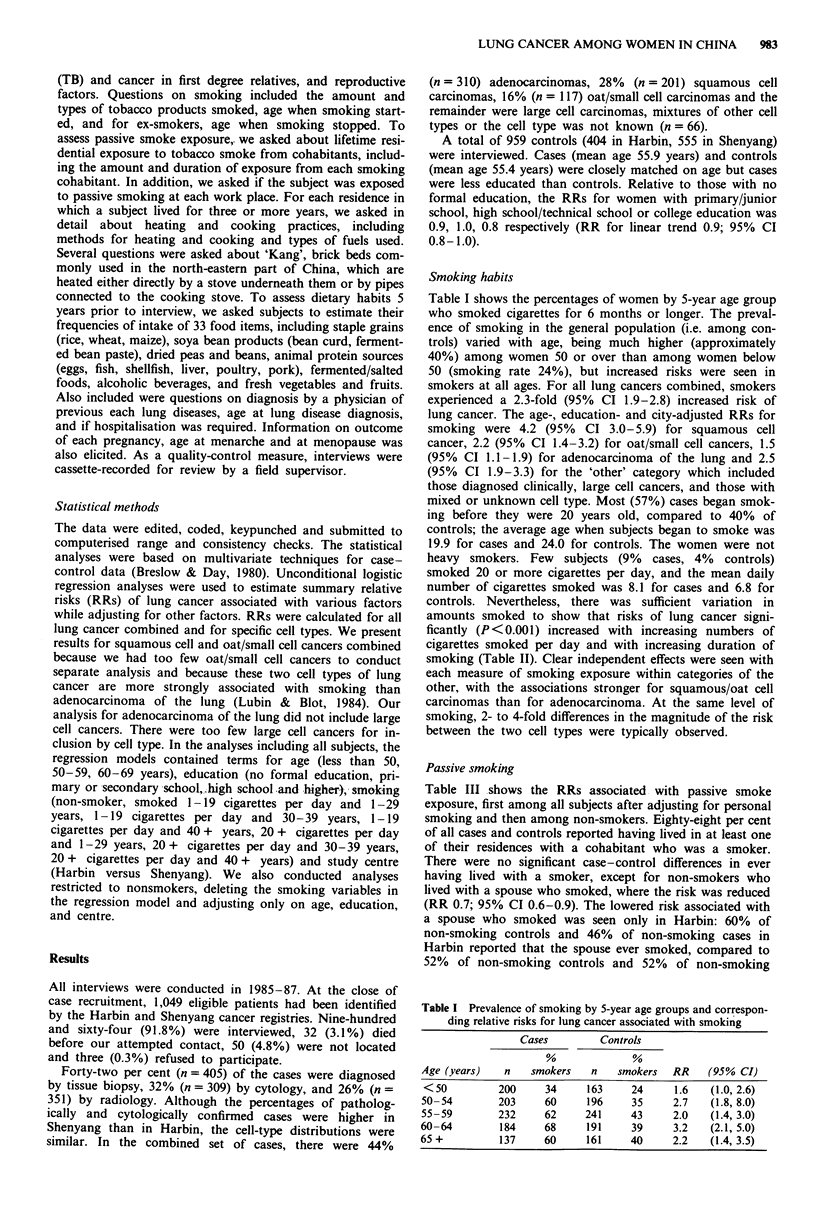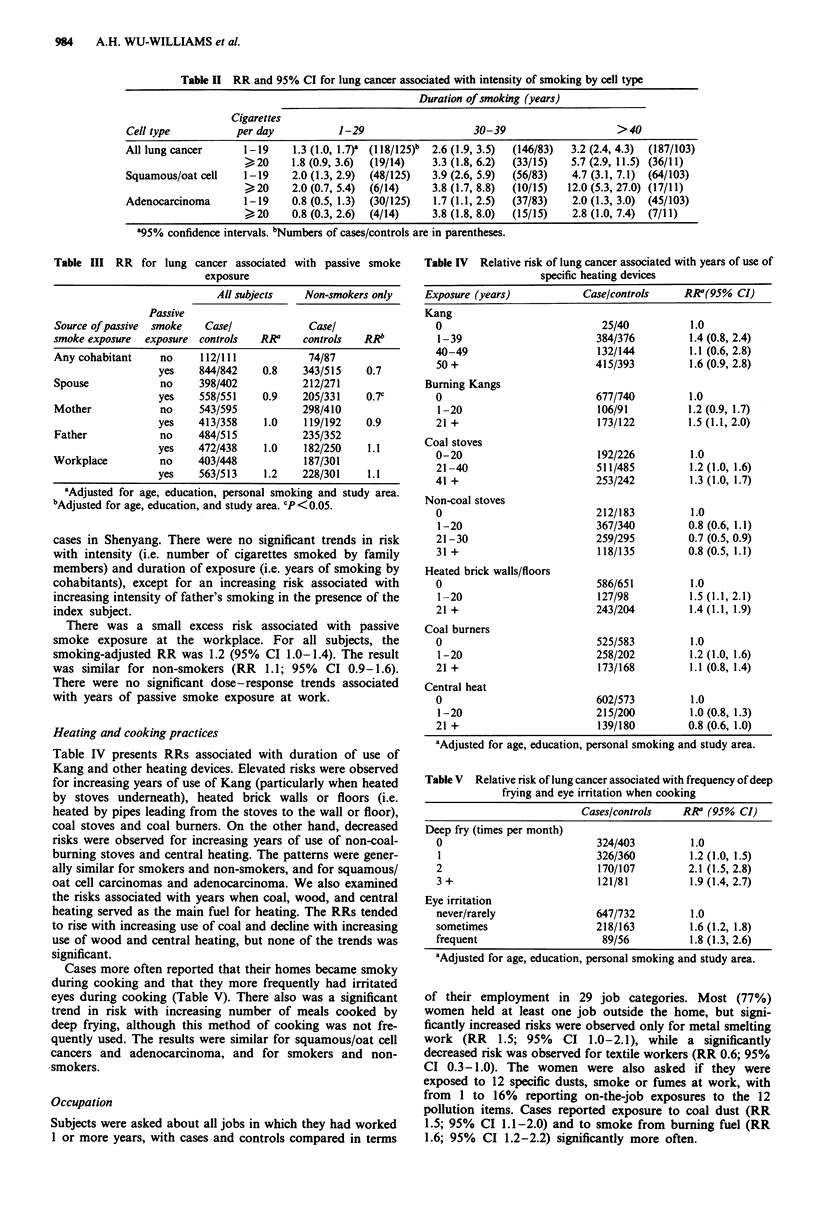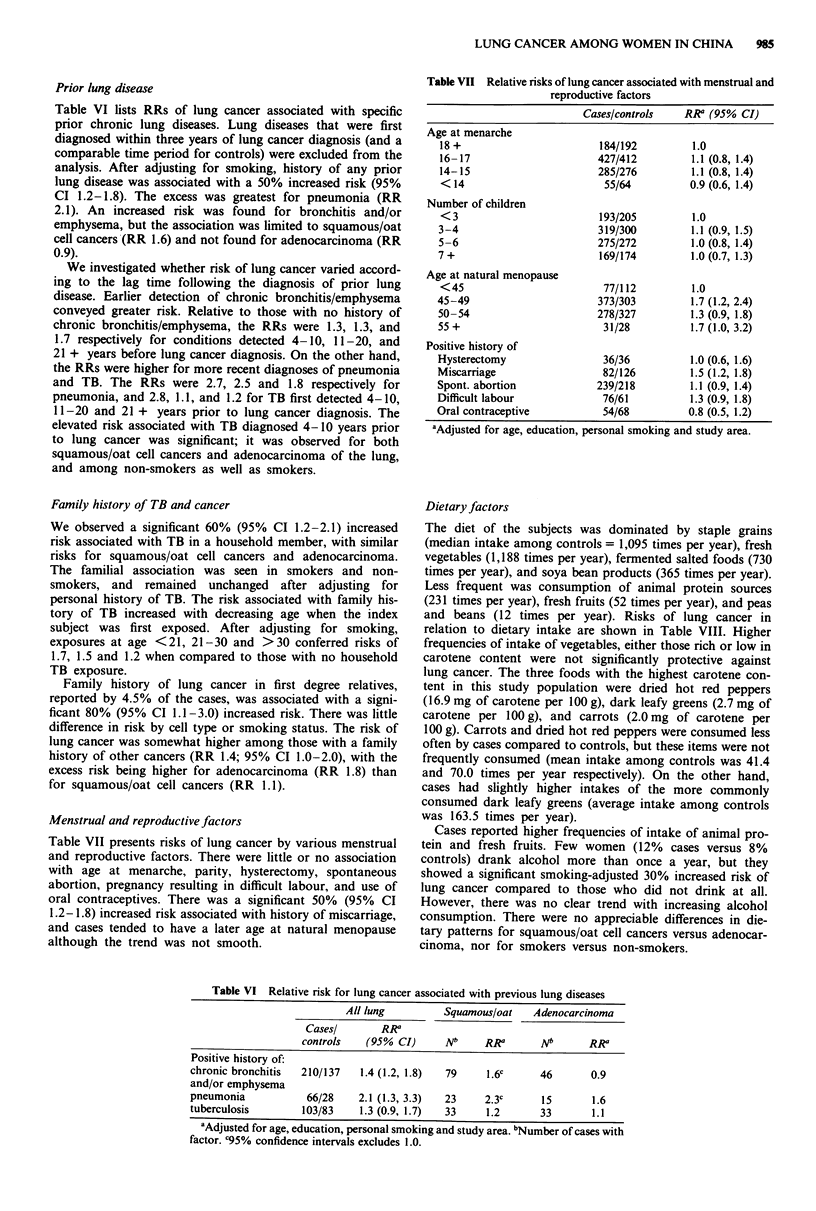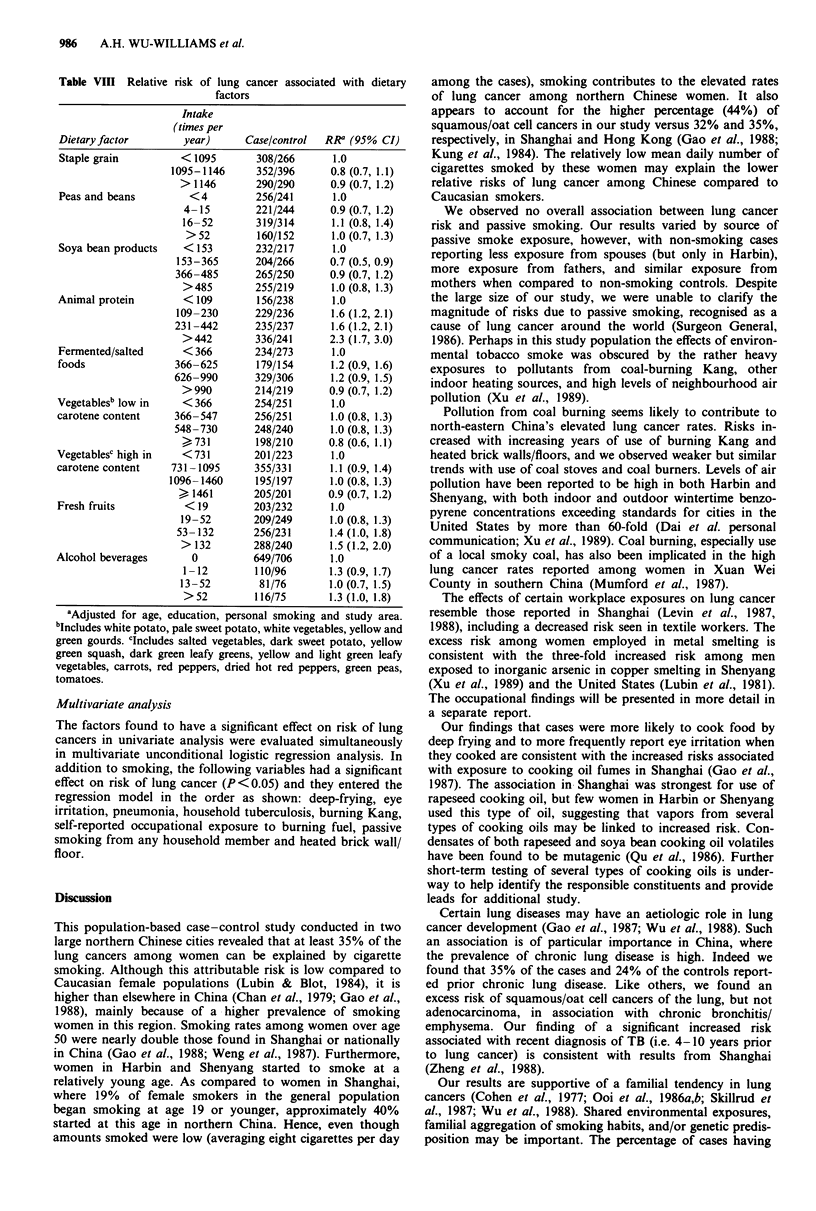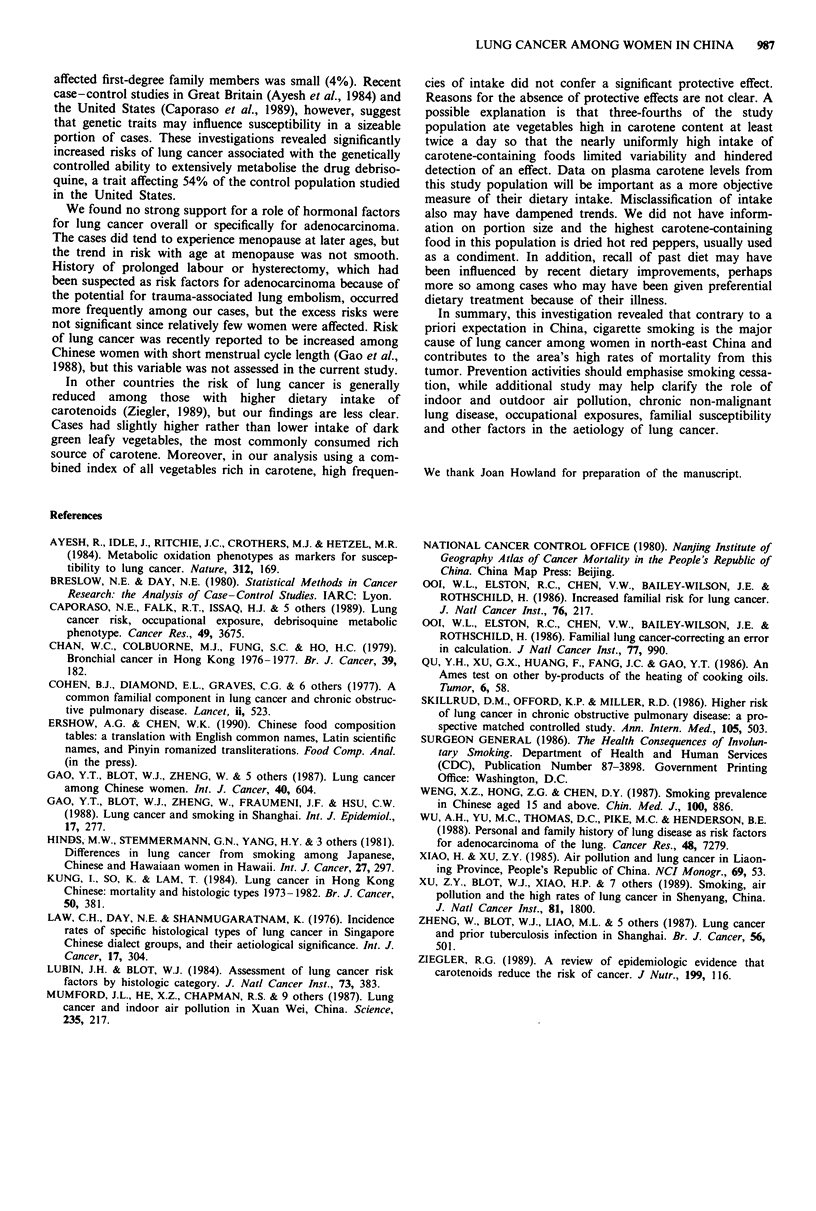# Lung cancer among women in north-east China.

**DOI:** 10.1038/bjc.1990.421

**Published:** 1990-12

**Authors:** A. H. Wu-Williams, X. D. Dai, W. Blot, Z. Y. Xu, X. W. Sun, H. P. Xiao, B. J. Stone, S. F. Yu, Y. P. Feng, A. G. Ershow

**Affiliations:** Department of Preventive Medicine, University of Southern California School of Medicine, Los Angeles 90033.

## Abstract

A case-control study of lung cancer involving interviews with 965 female patients and 959 controls in Shenyang and Harbin, two industrial cities which have among the highest rates of lung cancer in China, revealed that cigarette smoking is the main causal factor and accounted for about 35% of the tumours among women. Although the amount smoked was low (the cases averaged eight cigarettes per day), the percentage of smokers among women over age 50 in these cities was nearly double the national average. Air pollution from coal burning stoves was implicated, as risks of lung cancer increased in proportion to years of exposure to 'Kang' and other heating devices indigenous to the region. In addition, the number of meals cooked by deep frying and the frequency of smokiness during cooking were associated with risk of lung cancer. More cases than controls reported workplace exposures to coal dust and to smoke from burning fuel. Elevated risks were observed for smelter workers and decreased risks for textile workers. Prior chronic bronchitis/emphysema, pneumonia, and recent tuberculosis contributed significantly to lung cancer risk, as did a history of tuberculosis and lung cancer in family members. Higher intake of carotene-rich vegetables was not protective against lung cancer in this population. The findings were qualitatively similar across the major cell types of lung cancer, except that the associations with smoking and previous lung diseases were stronger for squamous/oat cell cancers than for adenocarcinoma of the lung.


Br. J. Cancer (1990), 62, 982–987 © Macmillan Press Ltd., 1990

### Lung cancer among women in north-east China

A.H. Wu-Williams[1], X.D. Dai[3], W. Blot[2], Z.Y. Xu[4], X.W. Sun[3], H.P. Xiao[4], B.J. Stone[2], S.F. Yu[3], Y.P. Feng[4], A.G. Ershow[2], J. Sun[4], J.F. Fraumeni Jr[2] & B.E. Henderson[1]

[1]*Department of Preventive Medicine, University of Southern California School of Medicine, Los Angeles, CA 90033, USA;* [2]*National Cancer Institute, Bethesda, MD 20892, USA;* [3]*Harbin Medical School, Harbin, Heilongjiang Province, People's Republic of China; and* [4]*Liaoning Public Health and Anti-Epidemic Station, Shenyang, Liaoning Province, People's Republic of China.*

**Summary** A case-control study of lung cancer involving interviews with 965 female patients and 959 controls in Shenyang and Harbin, two industrial cities which have among the highest rates of lung cancer in China, revealed that cigarette smoking is the main causal factor and accounted for about 35% of the tumours among women. Although the amount smoked was low (the cases averaged eight cigarettes per day), the percentage of smokers among women over age 50 in these cities was nearly double the national average. Air pollution from coal burning stoves was implicated, as risks of lung cancer increased in proportion to years of exposure to 'Kang' and other heating devices indigenous to the region. In addition, the number of meals cooked by deep frying and the frequency of smokiness during cooking were associated with risk of lung cancer. More cases than controls reported workplace exposures to coal dust and to smoke from burning fuel. Elevated risks were observed for smelter workers and decreased risks for textile workers. Prior chronic bronchitis/emphysema, pneumonia, and recent tuberculosis contributed significantly to lung cancer risk, as did a history of tuberculosis and lung cancer in family members. Higher intake of carotene-rich vegetables was not protective against lung cancer in this population. The findings were qualitatively similar across the major cell types of lung cancer, except that the associations with smoking and previous lung diseases were stronger for squamous/oat cell cancers than for adenocarcinoma of the lung.

The rate of lung cancer among Chinese females is among the highest in the world. Elevated incidence, particularly of adenocarcinoma of the lung, has been noted for Chinese females residing in different geographic areas, including Singapore (Law *et al.*, 1976), Hong Kong (Kung *et al.*, 1984), Shanghai (Gao *et al.*, 1988) and the United States (Hinds *et al.*, 1981). The high rates are unusual because few Chinese women smoke tobacco. Within China, elevated rates of female lung cancer are found in urban areas such as Shanghai and in rural as well as urban areas of the northeastern provinces of Liaoning and Heilongjiang (National Cancer Control Office, 1980; Xu *et al.*, 1986). Reasons for the geographic variation and clustering of high rates of lung cancer in the northern provinces are not known. We report here the results from case-control studies conducted in Shenyang and Harbin, the two major industrial cities in Liaoning and Heilonjiang provinces, to evaluate the role of several potential risk factors.

### Methods

In 1985–87, investigators from the Liaoning Province Public Health and Anti-Epidemic Station and the US National Cancer Institute conducted a large lung cancer study including 1,517 males (729 cases, 788 controls) and 1,073 females (518 cases, 555 controls). During the same time period, investigators at Harbin Medical College and the University of Southern California conducted a case-control study focused on female lung cancer (446 cases, 404 controls). Investigators from both studies met during the planning phase of the study and adopted a unified protocol to ascertain and select cases and controls, and a common questionnaire for the interview component of the study. Data on risks from smoking and air pollution among men and women in Shenyang have been published elsewhere (Xu *et al.*, 1989). Herein we report risks among females associated with a variety of factors, increasing sample sizes by nearly 80% by combining information from the two cities.

### *Case ascertainment*

We sought to enrol all newly diagnosed primary lung cancers in females in the study areas between 1985 and 1987. Utilising the cancer registries of Harbin and Shenyang, a system of rapid case ascertainment was established with the cooperation of all the major hospitals serving its area (about 35 in each city). In brief, the admitting physicians at each participating hospital completed a case abstract form whenever a lung cancer was diagnosed. We received these abstracts on a bi-weekly basis and selected as eligible cases those with primary, incident lung cancers diagnosed among female residents of the study area who were aged less than 70 years at the time of diagnosis. The lung cancer diagnosis and cell-type classification were verified locally in each study area by a panel of pulmonary specialists and pathologists.

### *Control selection*

Controls were females randomly selected from the general populations of Harbin and Shenyang. Controls were frequently matched by 5-year age group to the expected distribution of cases, which was determined in advance using the number and age distribution of female lung cancer cases reported in the two cities in 1983. A three-stage sampling procedure was used to select each control. The initial unit for randomisation was the neighbourhood committee, of which there are about 1,500 each in Harbin and in Shenyang. Committees were randomly selected with replacement after weighting by their population sizes. Then we randomly chose a household group from the approximately 10–25 household groups within each selected neighbourhood committee. In the final stage, among all females in the 5-year age category within the household group, one was randomly selected.

### *Questionnaire*

A structured, pre-coded questionnaire was used by trained interviewers who conducted personal interviews with the participants in their homes or work sites or in the hospital/clinic. The interview gathered information on demographic factors, active and passive smoke exposure, lifetime residential and occupational histories, diet and cooking practices, personal history of nonmalignant lung diseases, history of tuberculosis

Correspondence: A.H. Wu-Williams.
Received 27 March 1990; and in revised form 25 July 1990.

(TB) and cancer in first degree relatives, and reproductive factors. Questions on smoking included the amount and types of tobacco products smoked, age when smoking started, and for ex-smokers, age when smoking stopped. To assess passive smoke exposure, we asked about lifetime residential exposure to tobacco smoke from cohabitants, including the amount and duration of exposure from each smoking cohabitant. In addition, we asked if the subject was exposed to passive smoking at each work place. For each residence in which a subject lived for three or more years, we asked in detail about heating and cooking practices, including methods for heating and cooking and types of fuels used. Several questions were asked about 'Kang', brick beds commonly used in the north-eastern part of China, which are heated either directly by a stove underneath them or by pipes connected to the cooking stove. To assess dietary habits 5 years prior to interview, we asked subjects to estimate their frequencies of intake of 33 food items, including staple grains (rice, wheat, maize), soya bean products (bean curd, fermented bean paste), dried peas and beans, animal protein sources (eggs, fish, shellfish, liver, poultry, pork), fermented/salted foods, alcoholic beverages, and fresh vegetables and fruits. Also included were questions on diagnosis by a physician of previous each lung diseases, age at lung disease diagnosis, and if hospitalisation was required. Information on outcome of each pregnancy, age at menarche and at menopause was also elicited. As a quality-control measure, interviews were cassette-recorded for review by a field supervisor.

*Statistical methods*

The data were edited, coded, keypunched and submitted to computerised range and consistency checks. The statistical analyses were based on multivariate techniques for case-control data (Breslow & Day, 1980). Unconditional logistic regression analyses were used to estimate summary relative risks (RRs) of lung cancer associated with various factors while adjusting for other factors. RRs were calculated for all lung cancer combined and for specific cell types. We present results for squamous cell and oat/small cell cancers combined because we had too few oat/small cell cancers to conduct separate analysis and because these two cell types of lung cancer are more strongly associated with smoking than adenocarcinoma of the lung (Lubin & Blot, 1984). Our analysis for adenocarcinoma of the lung did not include large cell cancers. There were too few large cell cancers for inclusion by cell type. In the analyses including all subjects, the regression models contained terms for age (less than 50, 50-59, 60-69 years), education (no formal education, primary or secondary school, high school and higher), smoking (non-smoker, smoked 1-19 cigarettes per day and 1-29 years, 1-19 cigarettes per day and 30-39 years, 1-19 cigarettes per day and 40+ years, 20+ cigarettes per day and 1-29 years, 20+ cigarettes per day and 30-39 years, 20+ cigarettes per day and 40+ years) and study centre (Harbin versus Shenyang). We also conducted analyses restricted to nonsmokers, deleting the smoking variables in the regression model and adjusting only on age, education, and centre.

### Results

All interviews were conducted in 1985-87. At the close of case recruitment, 1,049 eligible patients had been identified by the Harbin and Shenyang cancer registries. Nine-hundred and sixty-four (91.8%) were interviewed, 32 (3.1%) died before our attempted contact, 50 (4.8%) were not located and three (0.3%) refused to participate.

Forty-two per cent ($n = 405$) of the cases were diagnosed by tissue biopsy, 32% ($n = 309$) by cytology, and 26% ($n = 351$) by radiology. Although the percentages of pathologically and cytologically confirmed cases were higher in Shenyang than in Harbin, the cell-type distributions were similar. In the combined set of cases, there were 44% ($n = 310$) adenocarcinomas, 28% ($n = 201$) squamous cell carcinomas, 16% ($n = 117$) oat/small cell carcinomas and the remainder were large cell carcinomas, mixtures of other cell types or the cell type was not known ($n = 66$).

A total of 959 controls (404 in Harbin, 555 in Shenyang) were interviewed. Cases (mean age 55.9 years) and controls (mean age 55.4 years) were closely matched on age but cases were less educated than controls. Relative to those with no formal education, the RRs for women with primary/junior school, high school/technical school or college education was 0.9, 1.0, 0.8 respectively (RR for linear trend 0.9; 95% CI 0.8-1.0).

*Smoking habits*

Table I shows the percentages of women by 5-year age group who smoked cigarettes for 6 months or longer. The prevalence of smoking in the general population (i.e. among controls) varied with age, being much higher (approximately 40%) among women 50 or over than among women below 50 (smoking rate 24%), but increased risks were seen in smokers at all ages. For all lung cancers combined, smokers experienced a 2.3-fold (95% CI 1.9-2.8) increased risk of lung cancer. The age-, education- and city-adjusted RRs for smoking were 4.2 (95% CI 3.0-5.9) for squamous cell cancer, 2.2 (95% CI 1.4-3.2) for oat/small cell cancers, 1.5 (95% CI 1.1-1.9) for adenocarcinoma of the lung and 2.5 (95% CI 1.9-3.3) for the 'other' category which included those diagnosed clinically, large cell cancers, and those with mixed or unknown cell type. Most (57%) cases began smoking before they were 20 years old, compared to 40% of controls; the average age when subjects began to smoke was 19.9 for cases and 24.0 for controls. The women were not heavy smokers. Few subjects (9% cases, 4% controls) smoked 20 or more cigarettes per day, and the mean daily number of cigarettes smoked was 8.1 for cases and 6.8 for controls. Nevertheless, there was sufficient variation in amounts smoked to show that risks of lung cancer significantly ($P < 0.001$) increased with increasing numbers of cigarettes smoked per day and with increasing duration of smoking (Table II). Clear independent effects were seen with each measure of smoking exposure within categories of the other, with the associations stronger for squamous/oat cell carcinomas than for adenocarcinoma. At the same level of smoking, 2- to 4-fold differences in the magnitude of the risk between the two cell types were typically observed.

*Passive smoking*

Table III shows the RRs associated with passive smoke exposure, first among all subjects after adjusting for personal smoking and then among non-smokers. Eighty-eight per cent of all cases and controls reported having lived in at least one of their residences with a cohabitant who was a smoker. There were no significant case-control differences in ever having lived with a smoker, except for non-smokers who lived with a spouse who smoked, where the risk was reduced (RR 0.7; 95% CI 0.6-0.9). The lowered risk associated with a spouse who smoked was seen only in Harbin: 60% of non-smoking controls and 46% of non-smoking cases in Harbin reported that the spouse ever smoked, compared to 52% of non-smoking controls and 52% of non-smoking

**Table I** Prevalence of smoking by 5-year age groups and corresponding relative risks for lung cancer associated with smoking

| | Cases | | Controls | | | |
|---|---|---|---|---|---|---|
| *Age (years)* | *n* | *% smokers* | *n* | *% smokers* | *RR* | *(95% CI)* |
| <50 | 200 | 34 | 163 | 24 | 1.6 | (1.0, 2.6) |
| 50-54 | 203 | 60 | 196 | 35 | 2.7 | (1.8, 8.0) |
| 55-59 | 232 | 62 | 241 | 43 | 2.0 | (1.4, 3.0) |
| 60-64 | 184 | 68 | 191 | 39 | 3.2 | (2.1, 5.0) |
| 65+ | 137 | 60 | 161 | 40 | 2.2 | (1.4, 3.5) |

**Table II** RR and 95% CI for lung cancer associated with intensity of smoking by cell type

| | | *Duration of smoking (years)* | | | | | |
|---|---|---|---|---|---|---|---|
| *Cell type* | *Cigarettes per day* | *1–29* | | *30–39* | | *>40* | |
| All lung cancer | 1–19 | 1.3 (1.0, 1.7)[a] | (118/125)[b] | 2.6 (1.9, 3.5) | (146/83) | 3.2 (2.4, 4.3) | (187/103) |
| | ⩾20 | 1.8 (0.9, 3.6) | (19/14) | 3.3 (1.8, 6.2) | (33/15) | 5.7 (2.9, 11.5) | (36/11) |
| Squamous/oat cell | 1–19 | 2.0 (1.3, 2.9) | (48/125) | 3.9 (2.6, 5.9) | (56/83) | 4.7 (3.1, 7.1) | (64/103) |
| | ⩾20 | 2.0 (0.7, 5.4) | (6/14) | 3.8 (1.7, 8.8) | (10/15) | 12.0 (5.3, 27.0) | (17/11) |
| Adenocarcinoma | 1–19 | 0.8 (0.5, 1.3) | (30/125) | 1.7 (1.1, 2.5) | (37/83) | 2.0 (1.3, 3.0) | (45/103) |
| | ⩾20 | 0.8 (0.3, 2.6) | (4/14) | 3.8 (1.8, 8.0) | (15/15) | 2.8 (1.0, 7.4) | (7/11) |

[a]95% confidence intervals. [b]Numbers of cases/controls are in parentheses.

**Table III** RR for lung cancer associated with passive smoke exposure

| | | *All subjects* | | *Non-smokers only* | |
|---|---|---|---|---|---|
| *Source of passive smoke exposure* | *Passive smoke exposure* | *Case/ controls* | *RR[a]* | *Case/ controls* | *RR[b]* |
| Any cohabitant | no | 112/111 | | 74/87 | |
| | yes | 844/842 | 0.8 | 343/515 | 0.7 |
| Spouse | no | 398/402 | | 212/271 | |
| | yes | 558/551 | 0.9 | 205/331 | 0.7[c] |
| Mother | no | 543/595 | | 298/410 | |
| | yes | 413/358 | 1.0 | 119/192 | 0.9 |
| Father | no | 484/515 | | 235/352 | |
| | yes | 472/438 | 1.0 | 182/250 | 1.1 |
| Workplace | no | 403/448 | | 187/301 | |
| | yes | 563/513 | 1.2 | 228/301 | 1.1 |

[a]Adjusted for age, education, personal smoking and study area. [b]Adjusted for age, education, and study area. [c]$P < 0.05$.

cases in Shenyang. There were no significant trends in risk with intensity (i.e. number of cigarettes smoked by family members) and duration of exposure (i.e. years of smoking by cohabitants), except for an increasing risk associated with increasing intensity of father's smoking in the presence of the index subject.

There was a small excess risk associated with passive smoke exposure at the workplace. For all subjects, the smoking-adjusted RR was 1.2 (95% CI 1.0–1.4). The result was similar for non-smokers (RR 1.1; 95% CI 0.9–1.6). There were no significant dose–response trends associated with years of passive smoke exposure at work.

### *Heating and cooking practices*

Table IV presents RRs associated with duration of use of Kang and other heating devices. Elevated risks were observed for increasing years of use of Kang (particularly when heated by stoves underneath), heated brick walls or floors (i.e. heated by pipes leading from the stoves to the wall or floor), coal stoves and coal burners. On the other hand, decreased risks were observed for increasing years of use of non-coal-burning stoves and central heating. The patterns were generally similar for smokers and non-smokers, and for squamous/oat cell carcinomas and adenocarcinoma. We also examined the risks associated with years when coal, wood, and central heating served as the main fuel for heating. The RRs tended to rise with increasing use of coal and decline with increasing use of wood and central heating, but none of the trends was significant.

Cases more often reported that their homes became smoky during cooking and that they more frequently had irritated eyes during cooking (Table V). There also was a significant trend in risk with increasing number of meals cooked by deep frying, although this method of cooking was not frequently used. The results were similar for squamous/oat cell cancers and adenocarcinoma, and for smokers and non-smokers.

### *Occupation*

Subjects were asked about all jobs in which they had worked 1 or more years, with cases and controls compared in terms of their employment in 29 job categories. Most (77%) women held at least one job outside the home, but significantly increased risks were observed only for metal smelting work (RR 1.5; 95% CI 1.0–2.1), while a significantly decreased risk was observed for textile workers (RR 0.6; 95% CI 0.3–1.0). The women were also asked if they were exposed to 12 specific dusts, smoke or fumes at work, with from 1 to 16% reporting on-the-job exposures to the 12 pollution items. Cases reported exposure to coal dust (RR 1.5; 95% CI 1.1–2.0) and to smoke from burning fuel (RR 1.6; 95% CI 1.2–2.2) significantly more often.

**Table IV** Relative risk of lung cancer associated with years of use of specific heating devices

| *Exposure (years)* | *Case/controls* | *RR[a](95% CI)* |
|---|---|---|
| Kang | | |
| 0 | 25/40 | 1.0 |
| 1–39 | 384/376 | 1.4 (0.8, 2.4) |
| 40–49 | 132/144 | 1.1 (0.6, 2.8) |
| 50 + | 415/393 | 1.6 (0.9, 2.8) |
| Burning Kangs | | |
| 0 | 677/740 | 1.0 |
| 1–20 | 106/91 | 1.2 (0.9, 1.7) |
| 21 + | 173/122 | 1.5 (1.1, 2.0) |
| Coal stoves | | |
| 0–20 | 192/226 | 1.0 |
| 21–40 | 511/485 | 1.2 (1.0, 1.6) |
| 41 + | 253/242 | 1.3 (1.0, 1.7) |
| Non-coal stoves | | |
| 0 | 212/183 | 1.0 |
| 1–20 | 367/340 | 0.8 (0.6, 1.1) |
| 21–30 | 259/295 | 0.7 (0.5, 0.9) |
| 31 + | 118/135 | 0.8 (0.5, 1.1) |
| Heated brick walls/floors | | |
| 0 | 586/651 | 1.0 |
| 1–20 | 127/98 | 1.5 (1.1, 2.1) |
| 21 + | 243/204 | 1.4 (1.1, 1.9) |
| Coal burners | | |
| 0 | 525/583 | 1.0 |
| 1–20 | 258/202 | 1.2 (1.0, 1.6) |
| 21 + | 173/168 | 1.1 (0.8, 1.4) |
| Central heat | | |
| 0 | 602/573 | 1.0 |
| 1–20 | 215/200 | 1.0 (0.8, 1.3) |
| 21 + | 139/180 | 0.8 (0.6, 1.0) |

[a]Adjusted for age, education, personal smoking and study area.

**Table V** Relative risk of lung cancer associated with frequency of deep frying and eye irritation when cooking

| | *Cases/controls* | *RR[a] (95% CI)* |
|---|---|---|
| Deep fry (times per month) | | |
| 0 | 324/403 | 1.0 |
| 1 | 326/360 | 1.2 (1.0, 1.5) |
| 2 | 170/107 | 2.1 (1.5, 2.8) |
| 3 + | 121/81 | 1.9 (1.4, 2.7) |
| Eye irritation | | |
| never/rarely | 647/732 | 1.0 |
| sometimes | 218/163 | 1.6 (1.2, 1.8) |
| frequent | 89/56 | 1.8 (1.3, 2.6) |

[a]Adjusted for age, education, personal smoking and study area.

*Prior lung disease*

Table VI lists RRs of lung cancer associated with specific prior chronic lung diseases. Lung diseases that were first diagnosed within three years of lung cancer diagnosis (and a comparable time period for controls) were excluded from the analysis. After adjusting for smoking, history of any prior lung disease was associated with a 50% increased risk (95% CI 1.2–1.8). The excess was greatest for pneumonia (RR 2.1). An increased risk was found for bronchitis and/or emphysema, but the association was limited to squamous/oat cell cancers (RR 1.6) and not found for adenocarcinoma (RR 0.9).

We investigated whether risk of lung cancer varied according to the lag time following the diagnosis of prior lung disease. Earlier detection of chronic bronchitis/emphysema conveyed greater risk. Relative to those with no history of chronic bronchitis/emphysema, the RRs were 1.3, 1.3, and 1.7 respectively for conditions detected 4–10, 11–20, and 21 + years before lung cancer diagnosis. On the other hand, the RRs were higher for more recent diagnoses of pneumonia and TB. The RRs were 2.7, 2.5 and 1.8 respectively for pneumonia, and 2.8, 1.1, and 1.2 for TB first detected 4–10, 11–20 and 21 + years prior to lung cancer diagnosis. The elevated risk associated with TB diagnosed 4–10 years prior to lung cancer was significant; it was observed for both squamous/oat cell cancers and adenocarcinoma of the lung, and among non-smokers as well as smokers.

**Table VII** Relative risks of lung cancer associated with menstrual and reproductive factors

| | *Cases/controls* | *RR[a] (95% CI)* |
|---|---|---|
| Age at menarche | | |
| 18 + | 184/192 | 1.0 |
| 16–17 | 427/412 | 1.1 (0.8, 1.4) |
| 14–15 | 285/276 | 1.1 (0.8, 1.4) |
| <14 | 55/64 | 0.9 (0.6, 1.4) |
| Number of children | | |
| <3 | 193/205 | 1.0 |
| 3–4 | 319/300 | 1.1 (0.9, 1.5) |
| 5–6 | 275/272 | 1.0 (0.8, 1.4) |
| 7 + | 169/174 | 1.0 (0.7, 1.3) |
| Age at natural menopause | | |
| <45 | 77/112 | 1.0 |
| 45–49 | 373/303 | 1.7 (1.2, 2.4) |
| 50–54 | 278/327 | 1.3 (0.9, 1.8) |
| 55 + | 31/28 | 1.7 (1.0, 3.2) |
| Positive history of | | |
| Hysterectomy | 36/36 | 1.0 (0.6, 1.6) |
| Miscarriage | 82/126 | 1.5 (1.2, 1.8) |
| Spont. abortion | 239/218 | 1.1 (0.9, 1.4) |
| Difficult labour | 76/61 | 1.3 (0.9, 1.8) |
| Oral contraceptive | 54/68 | 0.8 (0.5, 1.2) |

[a]Adjusted for age, education, personal smoking and study area.

*Family history of TB and cancer*

We observed a significant 60% (95% CI 1.2–2.1) increased risk associated with TB in a household member, with similar risks for squamous/oat cell cancers and adenocarcinoma. The familial association was seen in smokers and non-smokers, and remained unchanged after adjusting for personal history of TB. The risk associated with family history of TB increased with decreasing age when the index subject was first exposed. After adjusting for smoking, exposures at age <21, 21–30 and >30 conferred risks of 1.7, 1.5 and 1.2 when compared to those with no household TB exposure.

Family history of lung cancer in first degree relatives, reported by 4.5% of the cases, was associated with a significant 80% (95% CI 1.1–3.0) increased risk. There was little difference in risk by cell type or smoking status. The risk of lung cancer was somewhat higher among those with a family history of other cancers (RR 1.4; 95% CI 1.0–2.0), with the excess risk being higher for adenocarcinoma (RR 1.8) than for squamous/oat cell cancers (RR 1.1).

*Menstrual and reproductive factors*

Table VII presents risks of lung cancer by various menstrual and reproductive factors. There were little or no association with age at menarche, parity, hysterectomy, spontaneous abortion, pregnancy resulting in difficult labour, and use of oral contraceptives. There was a significant 50% (95% CI 1.2–1.8) increased risk associated with history of miscarriage, and cases tended to have a later age at natural menopause although the trend was not smooth.

*Dietary factors*

The diet of the subjects was dominated by staple grains (median intake among controls = 1,095 times per year), fresh vegetables (1,188 times per year), fermented salted foods (730 times per year), and soya bean products (365 times per year). Less frequent was consumption of animal protein sources (231 times per year), fresh fruits (52 times per year), and peas and beans (12 times per year). Risks of lung cancer in relation to dietary intake are shown in Table VIII. Higher frequencies of intake of vegetables, either those rich or low in carotene content were not significantly protective against lung cancer. The three foods with the highest carotene content in this study population were dried hot red peppers (16.9 mg of carotene per 100 g), dark leafy greens (2.7 mg of carotene per 100 g), and carrots (2.0 mg of carotene per 100 g). Carrots and dried hot red peppers were consumed less often by cases compared to controls, but these items were not frequently consumed (mean intake among controls was 41.4 and 70.0 times per year respectively). On the other hand, cases had slightly higher intakes of the more commonly consumed dark leafy greens (average intake among controls was 163.5 times per year).

Cases reported higher frequencies of intake of animal protein and fresh fruits. Few women (12% cases versus 8% controls) drank alcohol more than once a year, but they showed a significant smoking-adjusted 30% increased risk of lung cancer compared to those who did not drink at all. However, there was no clear trend with increasing alcohol consumption. There were no appreciable differences in dietary patterns for squamous/oat cell cancers versus adenocarcinoma, nor for smokers versus non-smokers.

**Table VI** Relative risk for lung cancer associated with previous lung diseases

| | *All lung* | | *Squamous/oat* | | *Adenocarcinoma* | |
|---|---|---|---|---|---|---|
| | *Cases/ controls* | *RR[a] (95% CI)* | *N[b]* | *RR[a]* | *N[b]* | *RR[a]* |
| Positive history of: | | | | | | |
| chronic bronchitis and/or emphysema | 210/137 | 1.4 (1.2, 1.8) | 79 | 1.6[c] | 46 | 0.9 |
| pneumonia | 66/28 | 2.1 (1.3, 3.3) | 23 | 2.3[c] | 15 | 1.6 |
| tuberculosis | 103/83 | 1.3 (0.9, 1.7) | 33 | 1.2 | 33 | 1.1 |

[a]Adjusted for age, education, personal smoking and study area. [b]Number of cases with factor. [c]95% confidence intervals excludes 1.0.

**Table VIII** Relative risk of lung cancer associated with dietary factors

| Dietary factor | Intake (times per year) | Case/control | RR[a] (95% CI) |
|---|---|---|---|
| Staple grain | <1095 | 308/266 | 1.0 |
| | 1095–1146 | 352/396 | 0.8 (0.7, 1.1) |
| | >1146 | 290/290 | 0.9 (0.7, 1.2) |
| Peas and beans | <4 | 256/241 | 1.0 |
| | 4–15 | 221/244 | 0.9 (0.7, 1.2) |
| | 16–52 | 319/314 | 1.1 (0.8, 1.4) |
| | >52 | 160/152 | 1.0 (0.7, 1.3) |
| Soya bean products | <153 | 232/217 | 1.0 |
| | 153–365 | 204/266 | 0.7 (0.5, 0.9) |
| | 366–485 | 265/250 | 0.9 (0.7, 1.2) |
| | >485 | 255/219 | 1.0 (0.8, 1.3) |
| Animal protein | <109 | 156/238 | 1.0 |
| | 109–230 | 229/236 | 1.6 (1.2, 2.1) |
| | 231–442 | 235/237 | 1.6 (1.2, 2.1) |
| | >442 | 336/241 | 2.3 (1.7, 3.0) |
| Fermented/salted foods | <366 | 234/273 | 1.0 |
| | 366–625 | 179/154 | 1.2 (0.9, 1.6) |
| | 626–990 | 329/306 | 1.2 (0.9, 1.5) |
| | >990 | 214/219 | 0.9 (0.7, 1.2) |
| Vegetables[b] low in carotene content | <366 | 254/251 | 1.0 |
| | 366–547 | 256/251 | 1.0 (0.8, 1.3) |
| | 548–730 | 248/240 | 1.0 (0.8, 1.3) |
| | ≥731 | 198/210 | 0.8 (0.6, 1.1) |
| Vegetables[c] high in carotene content | <731 | 201/223 | 1.0 |
| | 731–1095 | 355/331 | 1.1 (0.9, 1.4) |
| | 1096–1460 | 195/197 | 1.0 (0.8, 1.3) |
| | ≥1461 | 205/201 | 0.9 (0.7, 1.2) |
| Fresh fruits | <19 | 203/232 | 1.0 |
| | 19–52 | 209/249 | 1.0 (0.8, 1.3) |
| | 53–132 | 256/231 | 1.4 (1.0, 1.8) |
| | >132 | 288/240 | 1.5 (1.2, 2.0) |
| Alcohol beverages | 0 | 649/706 | 1.0 |
| | 1–12 | 110/96 | 1.3 (0.9, 1.7) |
| | 13–52 | 81/76 | 1.0 (0.7, 1.5) |
| | >52 | 116/75 | 1.3 (1.0, 1.8) |

[a]Adjusted for age, education, personal smoking and study area. [b]Includes white potato, pale sweet potato, white vegetables, yellow and green gourds. [c]Includes salted vegetables, dark sweet potato, yellow green squash, dark green leafy greens, yellow and light green leafy vegetables, carrots, red peppers, dried hot red peppers, green peas, tomatoes.

### *Multivariate analysis*

The factors found to have a significant effect on risk of lung cancers in univariate analysis were evaluated simultaneously in multivariate unconditional logistic regression analysis. In addition to smoking, the following variables had a significant effect on risk of lung cancer ($P<0.05$) and they entered the regression model in the order as shown: deep-frying, eye irritation, pneumonia, household tuberculosis, burning Kang, self-reported occupational exposure to burning fuel, passive smoking from any household member and heated brick wall/floor.

### Discussion

This population-based case–control study conducted in two large northern Chinese cities revealed that at least 35% of the lung cancers among women can be explained by cigarette smoking. Although this attributable risk is low compared to Caucasian female populations (Lubin & Blot, 1984), it is higher than elsewhere in China (Chan *et al.*, 1979; Gao *et al.*, 1988), mainly because of a higher prevalence of smoking women in this region. Smoking rates among women over age 50 were nearly double those found in Shanghai or nationally in China (Gao *et al.*, 1988; Weng *et al.*, 1987). Furthermore, women in Harbin and Shenyang started to smoke at a relatively young age. As compared to women in Shanghai, where 19% of female smokers in the general population began smoking at age 19 or younger, approximately 40% started at this age in northern China. Hence, even though amounts smoked were low (averaging eight cigarettes per day among the cases), smoking contributes to the elevated rates of lung cancer among northern Chinese women. It also appears to account for the higher percentage (44%) of squamous/oat cell cancers in our study versus 32% and 35%, respectively, in Shanghai and Hong Kong (Gao *et al.*, 1988; Kung *et al.*, 1984). The relatively low mean daily number of cigarettes smoked by these women may explain the lower relative risks of lung cancer among Chinese compared to Caucasian smokers.

We observed no overall association between lung cancer risk and passive smoking. Our results varied by source of passive smoke exposure, however, with non-smoking cases reporting less exposure from spouses (but only in Harbin), more exposure from fathers, and similar exposure from mothers when compared to non-smoking controls. Despite the large size of our study, we were unable to clarify the magnitude of risks due to passive smoking, recognised as a cause of lung cancer around the world (Surgeon General, 1986). Perhaps in this study population the effects of environmental tobacco smoke was obscured by the rather heavy exposures to pollutants from coal-burning Kang, other indoor heating sources, and high levels of neighbourhood air pollution (Xu *et al.*, 1989).

Pollution from coal burning seems likely to contribute to north-eastern China's elevated lung cancer rates. Risks increased with increasing years of use of burning Kang and heated brick walls/floors, and we observed weaker but similar trends with use of coal stoves and coal burners. Levels of air pollution have been reported to be high in both Harbin and Shenyang, with both indoor and outdoor wintertime benzopyrene concentrations exceeding standards for cities in the United States by more than 60-fold (Dai *et al.* personal communication; Xu *et al.*, 1989). Coal burning, especially use of a local smoky coal, has also been implicated in the high lung cancer rates reported among women in Xuan Wei County in southern China (Mumford *et al.*, 1987).

The effects of certain workplace exposures on lung cancer resemble those reported in Shanghai (Levin *et al.*, 1987, 1988), including a decreased risk seen in textile workers. The excess risk among women employed in metal smelting is consistent with the three-fold increased risk among men exposed to inorganic arsenic in copper smelting in Shenyang (Xu *et al.*, 1989) and the United States (Lubin *et al.*, 1981). The occupational findings will be presented in more detail in a separate report.

Our findings that cases were more likely to cook food by deep frying and to more frequently report eye irritation when they cooked are consistent with the increased risks associated with exposure to cooking oil fumes in Shanghai (Gao *et al.*, 1987). The association in Shanghai was strongest for use of rapeseed cooking oil, but few women in Harbin or Shenyang used this type of oil, suggesting that vapors from several types of cooking oils may be linked to increased risk. Condensates of both rapeseed and soya bean cooking oil volatiles have been found to be mutagenic (Qu *et al.*, 1986). Further short-term testing of several types of cooking oils is underway to help identify the responsible constituents and provide leads for additional study.

Certain lung diseases may have an aetiologic role in lung cancer development (Gao *et al.*, 1987; Wu *et al.*, 1988). Such an association is of particular importance in China, where the prevalence of chronic lung disease is high. Indeed we found that 35% of the cases and 24% of the controls reported prior chronic lung disease. Like others, we found an excess risk of squamous/oat cell cancers of the lung, but not adenocarcinoma, in association with chronic bronchitis/emphysema. Our finding of a significant increased risk associated with recent diagnosis of TB (i.e. 4–10 years prior to lung cancer) is consistent with results from Shanghai (Zheng *et al.*, 1988).

Our results are supportive of a familial tendency in lung cancers (Cohen *et al.*, 1977; Ooi *et al.*, 1986*a*,*b*; Skillrud *et al.*, 1987; Wu *et al.*, 1988). Shared environmental exposures, familial aggregation of smoking habits, and/or genetic predisposition may be important. The percentage of cases having affected first-degree family members was small (4%). Recent case–control studies in Great Britain (Ayesh *et al.*, 1984) and the United States (Caporaso *et al.*, 1989), however, suggest that genetic traits may influence susceptibility in a sizeable portion of cases. These investigations revealed significantly increased risks of lung cancer associated with the genetically controlled ability to extensively metabolise the drug debrisoquine, a trait affecting 54% of the control population studied in the United States.

We found no strong support for a role of hormonal factors for lung cancer overall or specifically for adenocarcinoma. The cases did tend to experience menopause at later ages, but the trend in risk with age at menopause was not smooth. History of prolonged labour or hysterectomy, which had been suspected as risk factors for adenocarcinoma because of the potential for trauma-associated lung embolism, occurred more frequently among our cases, but the excess risks were not significant since relatively few women were affected. Risk of lung cancer was recently reported to be increased among Chinese women with short menstrual cycle length (Gao *et al.*, 1988), but this variable was not assessed in the current study.

In other countries the risk of lung cancer is generally reduced among those with higher dietary intake of carotenoids (Ziegler, 1989), but our findings are less clear. Cases had slightly higher rather than lower intake of dark green leafy vegetables, the most commonly consumed rich source of carotene. Moreover, in our analysis using a combined index of all vegetables rich in carotene, high frequencies of intake did not confer a significant protective effect. Reasons for the absence of protective effects are not clear. A possible explanation is that three-fourths of the study population ate vegetables high in carotene content at least twice a day so that the nearly uniformly high intake of carotene-containing foods limited variability and hindered detection of an effect. Data on plasma carotene levels from this study population will be important as a more objective measure of their dietary intake. Misclassification of intake also may have dampened trends. We did not have information on portion size and the highest carotene-containing food in this population is dried hot red peppers, usually used as a condiment. In addition, recall of past diet may have been influenced by recent dietary improvements, perhaps more so among cases who may have been given preferential dietary treatment because of their illness.

In summary, this investigation revealed that contrary to a priori expectation in China, cigarette smoking is the major cause of lung cancer among women in north-east China and contributes to the area's high rates of mortality from this tumor. Prevention activities should emphasise smoking cessation, while additional study may help clarify the role of indoor and outdoor air pollution, chronic non-malignant lung disease, occupational exposures, familial susceptibility and other factors in the aetiology of lung cancer.

We thank Joan Howland for preparation of the manuscript.

**References**

AYESH, R., IDLE, J., RITCHIE, J.C., CROTHERS, M.J. & HETZEL, M.R. (1984). Metabolic oxidation phenotypes as markers for susceptibility to lung cancer. *Nature*, **312**, 169.

BRESLOW, N.E. & DAY, N.E. (1980). *Statistical Methods in Cancer Research: the Analysis of Case–Control Studies.* IARC: Lyon.

CAPORASO, N.E., FALK, R.T., ISSAQ, H.J. & 5 others (1989). Lung cancer risk, occupational exposure, debrisoquine metabolic phenotype. *Cancer Res.*, **49**, 3675.

CHAN, W.C., COLBUORNE, M.J., FUNG, S.C. & HO, H.C. (1979). Bronchial cancer in Hong Kong 1976–1977. *Br. J. Cancer*, **39**, 182.

COHEN, B.J., DIAMOND, E.L., GRAVES, C.G. & 6 others (1977). A common familial component in lung cancer and chronic obstructive pulmonary disease. *Lancet*, **ii**, 523.

ERSHOW, A.G. & CHEN, W.K. (1990). Chinese food composition tables: a translation with English common names, Latin scientific names, and Pinyin romanized transliterations. *Food Comp. Anal.* (in the press).

GAO, Y.T., BLOT, W.J., ZHENG, W. & 5 others (1987). Lung cancer among Chinese women. *Int. J. Cancer*, **40**, 604.

GAO, Y.T., BLOT, W.J., ZHENG, W., FRAUMENI, J.F. & HSU, C.W. (1988). Lung cancer and smoking in Shanghai. *Int. J. Epidemiol.*, **17**, 277.

HINDS, M.W., STEMMERMANN, G.N., YANG, H.Y. & 3 others (1981). Differences in lung cancer from smoking among Japanese, Chinese and Hawaiaan women in Hawaii. *Int. J. Cancer*, **27**, 297.

KUNG, I., SO, K. & LAM, T. (1984). Lung cancer in Hong Kong Chinese: mortality and histologic types 1973–1982. *Br. J. Cancer*, **50**, 381.

LAW, C.H., DAY, N.E. & SHANMUGARATNAM, K. (1976). Incidence rates of specific histological types of lung cancer in Singapore Chinese dialect groups, and their aetiological significance. *Int. J. Cancer*, **17**, 304.

LUBIN, J.H. & BLOT, W.J. (1984). Assessment of lung cancer risk factors by histologic category. *J. Natl Cancer Inst.*, **73**, 383.

MUMFORD, J.L., HE, X.Z., CHAPMAN, R.S. & 9 others (1987). Lung cancer and indoor air pollution in Xuan Wei, China. *Science*, **235**, 217.

NATIONAL CANCER CONTROL OFFICE (1980). *Nanjing Institute of Geography Atlas of Cancer Mortality in the People's Republic of China.* China Map Press: Beijing.

OOI, W.L., ELSTON, R.C., CHEN, V.W., BAILEY-WILSON, J.E. & ROTHSCHILD, H. (1986). Increased familial risk for lung cancer. *J. Natl Cancer Inst.*, **76**, 217.

OOI, W.L., ELSTON, R.C., CHEN, V.W., BAILEY-WILSON, J.E. & ROTHSCHILD, H. (1986). Familial lung cancer-correcting an error in calculation. *J Natl Cancer Inst.*, **77**, 990.

QU, Y.H., XU, G.X., HUANG, F., FANG, J.C. & GAO, Y.T. (1986). An Ames test on other by-products of the heating of cooking oils. *Tumor*, **6**, 58.

SKILLRUD, D.M., OFFORD, K.P. & MILLER, R.D. (1986). Higher risk of lung cancer in chronic obstructive pulmonary disease: a prospective matched controlled study. *Ann. Intern. Med.*, **105**, 503.

SURGEON GENERAL (1986). *The Health Consequences of Involuntary Smoking.* Department of Health and Human Services (CDC), Publication Number 87-3898. Government Printing Office: Washington, D.C.

WENG, X.Z., HONG, Z.G. & CHEN, D.Y. (1987). Smoking prevalence in Chinese aged 15 and above. *Chin. Med. J.*, **100**, 886.

WU, A.H., YU, M.C., THOMAS, D.C., PIKE, M.C. & HENDERSON, B.E. (1988). Personal and family history of lung disease as risk factors for adenocarcinoma of the lung. *Cancer Res.*, **48**, 7279.

XIAO, H. & XU, Z.Y. (1985). Air pollution and lung cancer in Liaoning Province, People's Republic of China. *NCI Monogr.*, **69**, 53.

XU, Z.Y., BLOT, W.J., XIAO, H.P. & 7 others (1989). Smoking, air pollution and the high rates of lung cancer in Shenyang, China. *J. Natl Cancer Inst.*, **81**, 1800.

ZHENG, W., BLOT, W.J., LIAO, M.L. & 5 others (1987). Lung cancer and prior tuberculosis infection in Shanghai. *Br. J. Cancer*, **56**, 501.

ZIEGLER, R.G. (1989). A review of epidemiologic evidence that carotenoids reduce the risk of cancer. *J Nutr.*, **199**, 116.